# An attenuated *Shigella* mutant lacking the RNA-binding protein Hfq provides cross-protection against *Shigella* strains of broad serotype

**DOI:** 10.1371/journal.pntd.0005728

**Published:** 2017-07-20

**Authors:** Jiro Mitobe, Ritam Sinha, Soma Mitra, Dhrubajyoti Nag, Noriko Saito, Ken Shimuta, Nobuo Koizumi, Hemanta Koley

**Affiliations:** 1 Department of Bacteriology I, National Institute of Infectious Diseases, Shinjuku, Tokyo, Japan; 2 Division of Bacteriology, National Institute of Cholera and Enteric Diseases, Beliaghata, Kolkata, India; 3 Laboratory of Electron Microscopy, National Institute of Infectious Diseases, Shinjuku, Tokyo, Japan; Oxford University Clinical Research Unit, VIET NAM

## Abstract

Few live attenuated vaccines protect against multiple serotypes of bacterial pathogen because host serotype-specific immune responses are limited to the serotype present in the vaccine strain. Here, immunization with a mutant of *Shigella flexneri* 2a protected guinea pigs against subsequent infection by *S*. *dysenteriae* type 1 and *S*. *sonnei* strains. This deletion mutant lacked the RNA-binding protein Hfq leading to increased expression of the type III secretion system via loss of regulation, resulting in attenuation of cell viability through repression of stress response sigma factors. Such increased antigen production and simultaneous attenuation were expected to elicit protective immunity against *Shigella* strains of heterologous serotypes. Thus, the vaccine potential of this mutant was tested in two guinea pig models of shigellosis. Animals vaccinated in the left eye showed fewer symptoms upon subsequent challenge via the right eye, and even survived subsequent intestinal challenge. In addition, oral vaccination effectively induced production of immunoglobulins without severe side effects, again protecting all animals against subsequent intestinal challenge with *S*. *dysenteriae* type 1 or *S*. *sonnei* strains. Antibodies against common virulence proteins and the O-antigen of *S*. *flexneri* 2a were detected by immunofluorescence microscopy. Reaction of antibodies with various strains, including enteroinvasive *Escherichia coli*, suggested that common virulence proteins induced protective immunity against a range of serotypes. Therefore, vaccination is expected to cover not only the most prevalent serotypes of *S*. *sonnei* and *S*. *flexneri* 2a, but also various *Shigella* strains, including *S*. *dysenteriae* type 1, which produces Shiga toxin.

## Author summary

An ideal vaccine should show a broad range of protective efficacy against all serotypes of a particular pathogen. Variations in the structural lipopolysaccharide “O”-antigen mean that it is difficult to induce protection against multiple serotype *Shigella* strains using a single serotype immunogen. Here, we examined vaccination effects of an attenuated *S*. *flexneri hfq* mutant strain overexpressing virulence proteins common to all *Shigella* serotypes, which was identified from our studies on temperature and osmotic-dependent regulation of the type III secretion system: an essential virulence machinery among *Shigella* and enteroinvasive *Escherichia coli* species. The deletion mutant lacked the RNA-binding protein Hfq, which led to increased expression of the type III secretion system via loss of regulation, resulting in attenuation of cell viability through repression of stress response sigma factors. Immunization with the *hfq* mutant of *S*. *flexneri* 2a induced systemic immunity and cross-protective efficacy against two different serotypes of *Shigella* strain: *S*. *dysenteriae* type 1 and. *S*. *sonnei*. Detection of anti-*Shigella* antibodies against various strains with different serotypes suggests that generation of antibodies specific for common virulence proteins affords cross-protection against *Shigella* strains of multiple serotypes.

## Introduction

Shigellosis is common worldwide. It is estimated that 164.7 million people are infected annually, resulting in 1.1 million deaths. About 70% of episodes and 60% of deaths involve children under 5 years-of-age [1]. In addition, growing antibiotic resistance [2] is a serious problem in all countries; this is compounded by the fact that so many people travel. Therefore, vaccines against shigellosis are being developed [3, 4].

*Shigella* strains comprise four subspecies: *S*. *dysenteriae*, *S*. *flexneri*, *S*. *sonnei*, and *S*. *boydii*. These are further divided into 50 distinct serotypes [1, 3] according to the immunogenicity of capsular lipopolysaccharide O-antigens. The World Health Organization has set *Shigella dysenteriae* type 1 (*Sd1*) as a primary target for control because this strain produces Shiga toxin, a neuro-cytotoxic agent that causes hemolytic uremic syndrome (HUS) [5]. At present, *S*. *sonnei* is the most prevalent strain in industrialized countries [1]; however, imported epidemics of *Sd1* with HUS have been reported [6]. A Global Enteric Multicenter study also indicated that *S*. *flexneri* (65.9%), particularly serotype 2a (20.2%), and *S*. *sonnei* (23.7%) are the most prevalent strains isolated from patients aged <60 months at four sites in Africa and three sites in Southeast Asia [7].

The pathogenesis of *Shigella* strains is dependent on virulence plasmids [8] encoding common virulence factors [8, 9] belonging to the type III secretion system (T3SS) [10]. The T3SS is a needle-like transporter complex expressed on the surface of bacteria [11]; the complex injects effector molecules, such as IpaBCDA proteins (All genes and proteins are listed in Table 1), into host cells to facilitate bacterial invasion. After propagation within the colonic epithelium, the bacteria spread via a mechanism involving the outer membrane protein VirG (IcsA) [12, 13].

**Table 1:** List of accession numbers.

	UniProtKB-ID	GenBank Accession number	
Hfq	P0A6X3 (HFQ_ECOLI)	BAA00644.1	D00743.1
RpoE	P0AGB6 (RPOE_ECOLI)	AAC75626.1	U37089.1
RpoS	P13445 (RPOS_ECOLI)	AAC75783.1	D13548.1
IpaB	P18011 (IPAB_SHIFL)	AAA26522.1	J04117.1
IpaC	P18012 (IPAB_SHIFL)	AAA26523.1	J04117.1
IpaD	P18013 (IPAB_SHIFL)	AAA26524.1	J04117.1
IpaA	P18010 (IPAB_SHIFL)	AAA26525.1	J04117.1
Spa32	P0A1K5 (SPAN_SHIFL)	AAA26542.1	M81458.1
InvE/VirB	P0A248 (VIRB_SHISO)	AAA26520.1	M33790.1
IcsA/VirG	Q7BCK4 (ICSA_SHIFL)	AAA26547.1	M22802.1
IcsP	O33641 (ICSP_SHIFL)	AAC45084.1	U73461.1
VirF	P0A2T1 (VIRF_SHIFL)	AAA26545.1	M29172.1

Many studies, including a prospective epidemiological surveillance study of a cohort of children in an endemic area, indicate that acquired immunity to shigellosis is O-antigen specific [14–16]; therefore, a majority of vaccine candidates have been developed to provide serotype-specific protection. The strategy was similar to that used to develop the practical pneumococcal and Hib vaccines, although immunization with inactivated cells or cellular components appeared insufficient, suggesting that some processes during the infection cycle are required for effective antigen presentation to the intestinal system. Such candidate *Shigella* vaccines comprise attenuated strains derived by mutation of *virG* and some metabolic genes [17–19]. However, a licensed vaccine is still not available because many candidates have failed to maintain a subtle balance between immunogenicity and safety [3, 4].

Few studies support “cross protection” against both homologous and heterologous serotypes of *Shigella* strain. However, early field studies suggested the possibility. An attenuated *S*. *flexneri* 2a strain T_32_–Istrati was developed in Romania by serial passage of the culture. Oral administration of an extreme dose (0.5–2.0×10^11^ colony forming units (cfu)) of live bacteria) to volunteers was well tolerated. A field study of 32,000 children and 500 adults conducted in 1976–1980 reported 81% protection against *S*. *flexneri* 2a, 89% protection against *S*. *sonnei*, and 88% protection against other *Shigella* species [20, 21]. Also, a Chinese study using the same strain (the study enrolled 5000 vaccinees and 5000 controls) reported 85% protection against homologous serotypes and 72% against heterologous serotypes [21, 22]. However, no rationale for the observed cross protection was provided and no follow-up studies have been reported.

Recent attempts to utilize common virulence proteins for immunization have also been reported. Intra-nasal immunization of a mouse model of pneumonia with recombinant T3SS effectors IpaB and IpaD along with a variant of a heat-labile toxin from *E*. *coli* (dmLT) protected against subsequent challenge with *S*. *sonnei* and *S*. *flexneri* [23]. Kim *et al*. showed that the C-terminal region of the outer membrane protein IcsP is common to all *Shigella* serotypes. Nasal immunization of a mouse model of pneumonia with a vaccine containing the C-terminal peptide of IcsP and dmLT provided cross protection (>60%) against *S*. *flexneri* 2a, *S*. *flexneri* 6, and *Sd1* [24].

Here, we report cross protection provided by a *S*. *flexneri* 2a-based vaccine candidate. The idea originated from our basic studies on regulation of T3SS expression. A mutant of *S*. *sonnei* harboring a deletion of the *hfq* gene encoding an RNA-binding protein lost the temperature- and osmotic-dependent regulation, showed increased production of T3SS and increased invasion into Hela cells. This occurred because expression of a T3SS regulator, *invE* (*virB*) [25, 26], was up-regulated via loss of regulation at the post-transcriptional level [27–29]. As observed for many other pathogens, loss of *hfq* results in attenuation [29] via repression of stress response regulators such as *rpoE* [30] and *rpoS* [31]. Attenuation via *hfq* mutation is also reported for an experimental vaccine against *Salmonella typhimurium*, but the study showed only homologous protection [32]. Increased antigen expression and simultaneous attenuation are expected to elicit protective immunity against *Shigella* strains of heterologous serotypes. Thus, we also characterized the *hfq* mutant by examining its potential effects as a vaccine. We performed two different challenge experiments to evaluate whether the *Δhfq* mutant provided protection against subsequent infection by *Shigella* strains of heterologous serotypes. In addition, we examined production of antibodies against various heterologous serotypes.

## Materials and methods

### Ethics statement

All experimental protocols were approved by the Animal Ethical Committees at the National Institute of Infectious Diseases (NIID) (No. 208123 and 209002) and National Institute of Cholera and Enteric Diseases (NICED) (No. Apo/80/06/05/2011) and conducted in accordance with Guidelines for the Proper Conduct of Animal Experiments (Scientific Council of Japan) and Guidelines for the Care and Use of Animals in Scientific Research (Indian National Science Academy).

### Statistical analysis

Data are presented as the mean standard deviation (SD). Statistically significant differences between individual groups were analyzed using an unpaired Student’s *t*-test. *p*<0.05 was considered significant.

### Animal experiments

**Fig 1:**
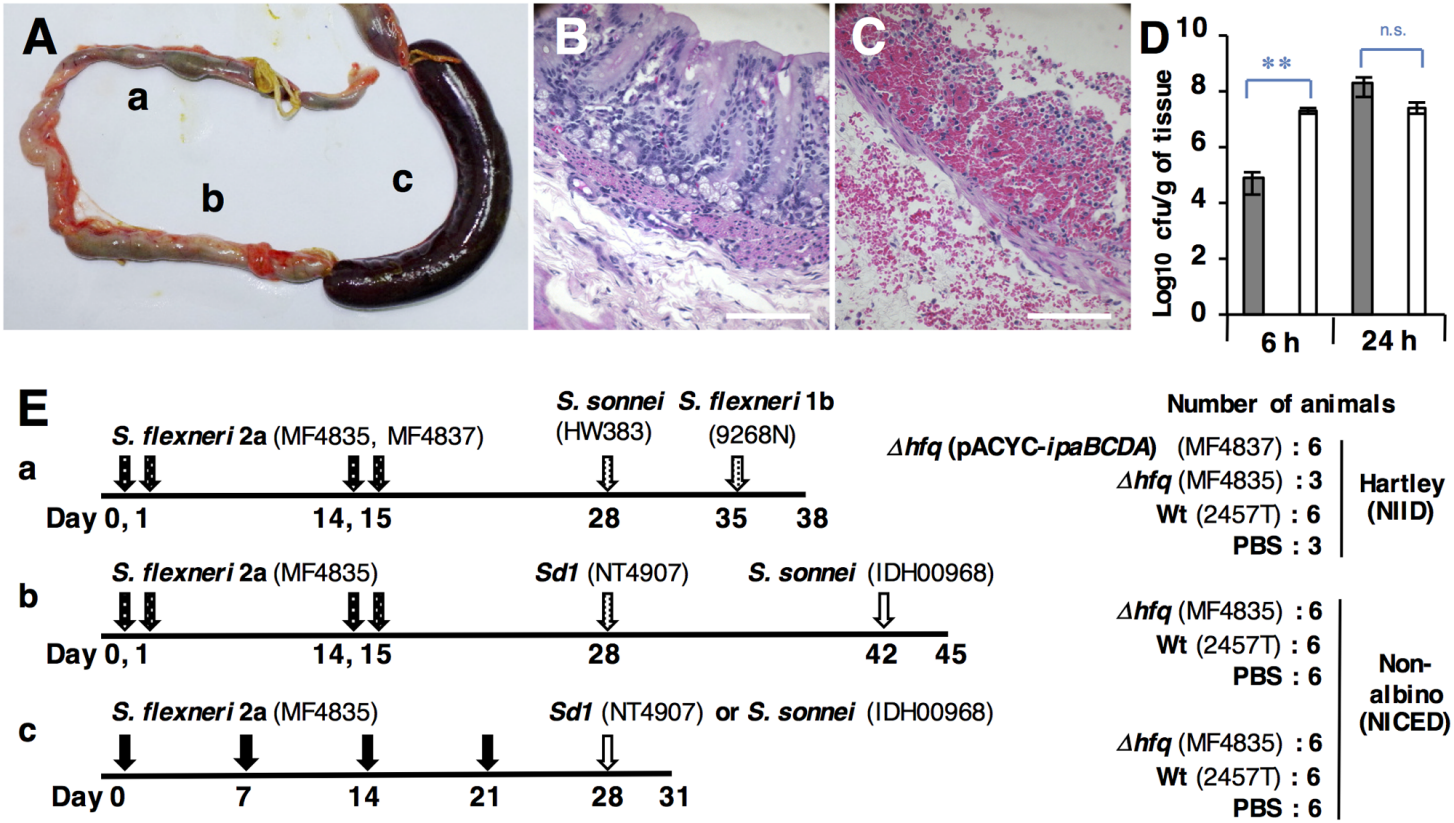
Characterization of the *Δhfq* mutant using a colon loop model, and the experimental schedules. (A) Colon segments were infected with (a) *Δhfq* (MF4835), (b) PBS, or (c) *S*. *flexneri* 2a Wt (2457T) for 24 h. Images of tissue infected with *Δhfq* (B) and Wt (C) for 24 h. Scale bars, 100 μm. (D) Bacterial counts within the tissue at 6 and 24 h post-inoculation. White and gray bars indicate *Δhfq* and Wt strains, respectively. Values are expressed as the mean ± SD; n = 3 animals. ** *p*<0.01; n.s., not significant. (E) Experimental schedules: (a) Ocular immunization/ocular challenge; (b) Ocular immunization/ocular/intestinal challenge; (c) Oral immunization/intestinal challenge. *Black* and *white arrowheads* denote immunization and challenge, respectively. Spotted arrowheads denote ocular inoculation.

**Fig 2:**
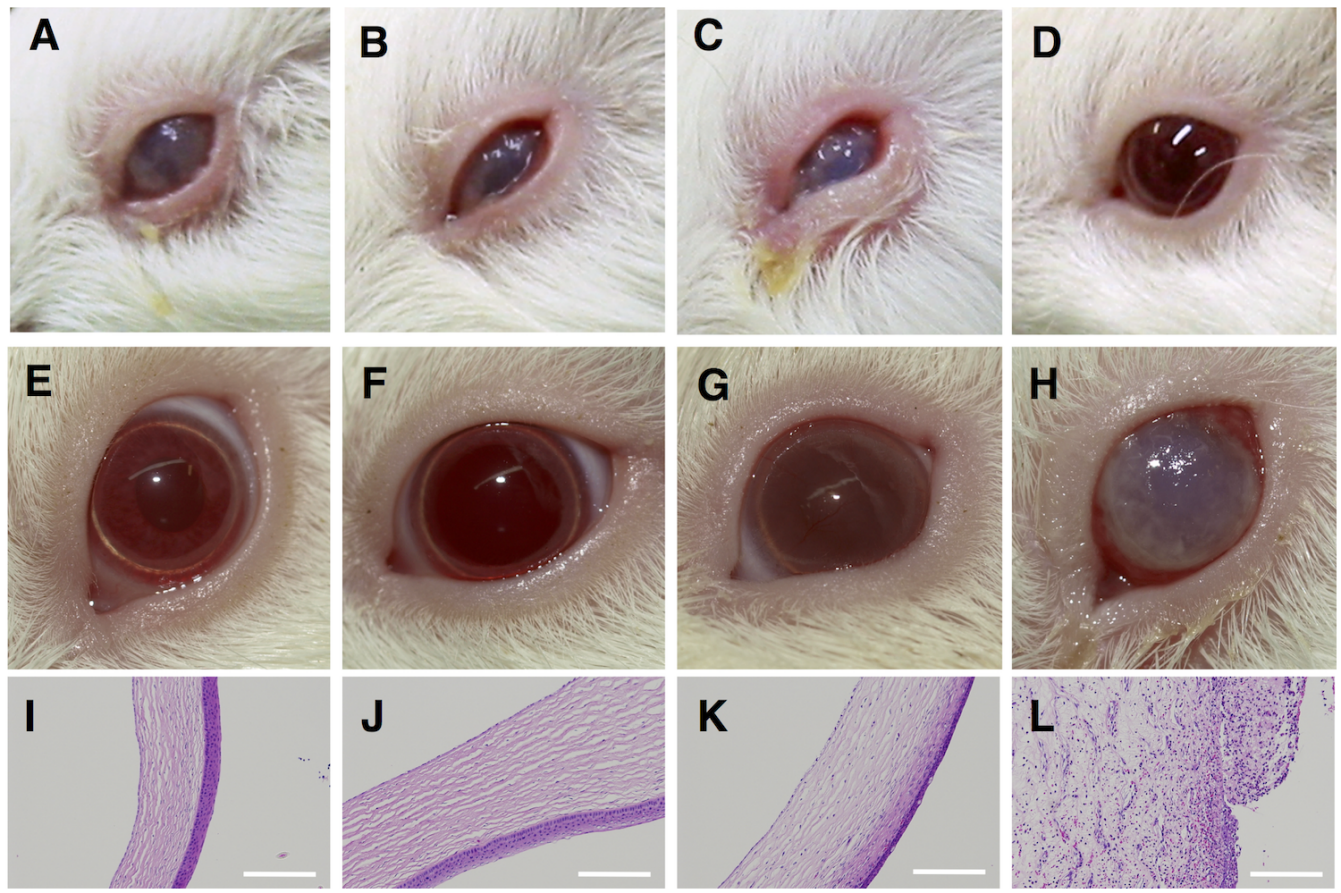
Ocular immunization and ocular challenge with *S*. *sonnei* strain (HW383). *Upper panels*: Representative eyes at 4 days post-immunization. *Middle panels*: Representative eyes at 4 days post-challenge with HW383. *Lower panels*: Sagittal sections of cornea at 3 days post-challenge with *S*. *flexneri* 1b (9268N). Scale bars, 200 μm. Animals were immunized with (A, E, I) *Δhfq* (MF4835), (B, F, J) *Δhfq* carrying the *ipaBCDA* plasmid (MF4837), (C, G, K) Wt strain (2457T), or (D, H, L) PBS.

**Fig 3:**
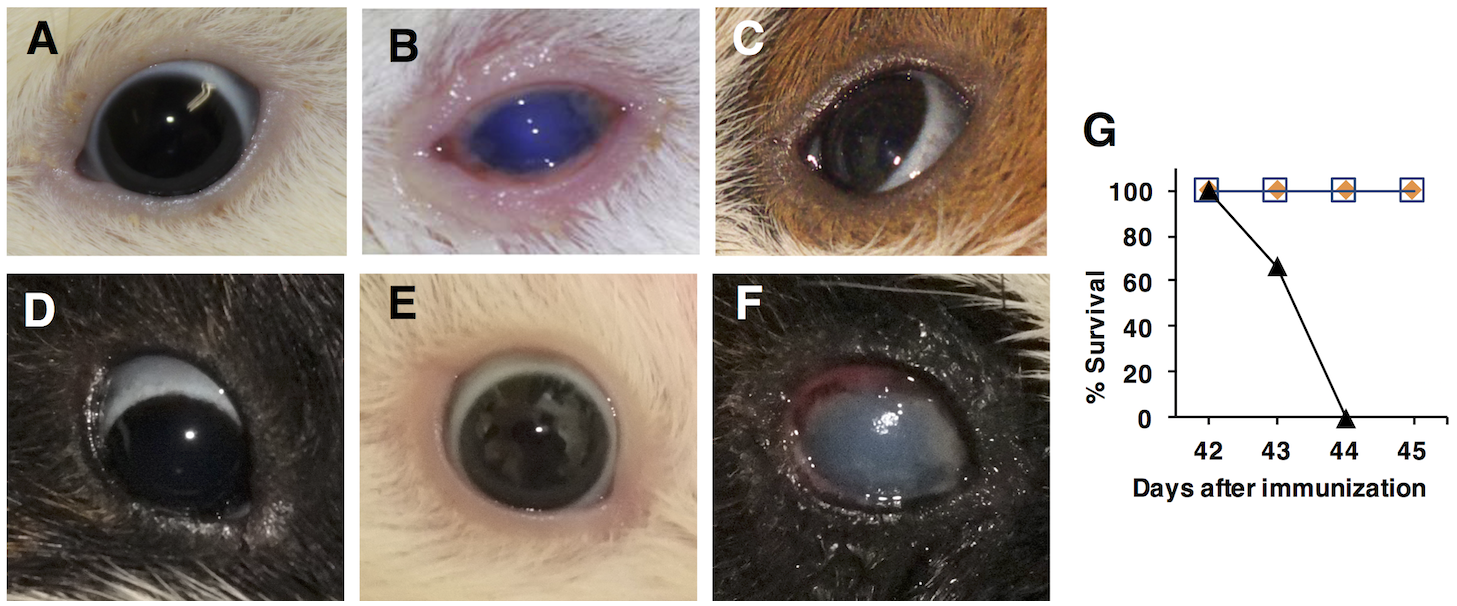
Ocular immunization and challenge with *Sd1* (NT4907), followed by intestinal challenge with *S*. *sonnei* (IDH00698). *Upper panels*: Left eyes of representative animals at 4 days post-immunization. *Lower panels*: Right eyes of the same animals at 4 days post-challenge with *Sd1* (NT4907). Animals were immunized with (A, D) *Δhfq* (MF4835), (B, E) Wt strain (2457T), or (C, F) PBS. (G) Survival curves after intestinal challenge. Symbols: blue square, *Δhfq*; orange diamond, Wt; black triangle, PBS.

**Fig 4:**
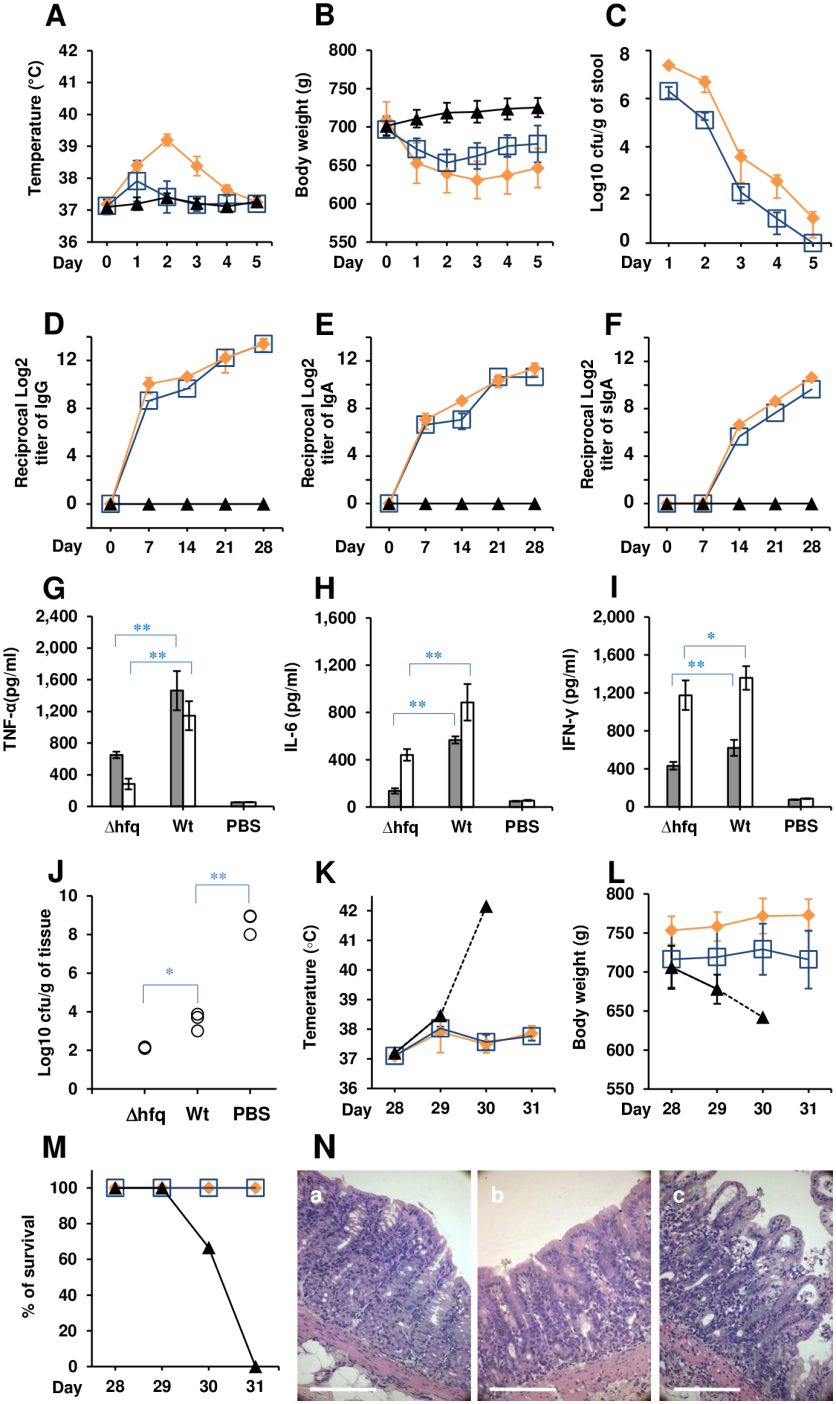
Oral immunization and intestinal challenge with *Sd1* (NT4907). Changes in (A) rectal temperature and (B) body weight. (C) Bacterial counts (immunizing strain) in stools. Levels of (D) IgG, (E) IgA, and (F) secretory IgA in stools. Symbols: blue square, *Δhfq*; orange diamond, Wt; black triangle, PBS. Levels of (G) TNF-α, (H) IL-6, and (I) IFN-γ in serum. Gray and white bars indicate values at Days 7 and 28 Days, respectively. Values are expressed as the mean ± SD; n = 6 animals. *p<0.05; **p<0.01. (J) Intestinal colonization in three animals at 24 h post-*Sd1* challenge. Changes in (K) rectal temperature and (L) body weight. Symbols: blue square, *Δhfq*; orange diamond, Wt; black triangle, PBS. Values are expressed as the mean ± SD; n = 3 animals. Values derived from fewer than three animals are indicated by a dashed line. (M) Survival curves. (N) Tissues from animals immunized with *Δhfq* (a), Wt strain (b), or PBS (c). Scale bars, 100 μm.

**Fig 5:**
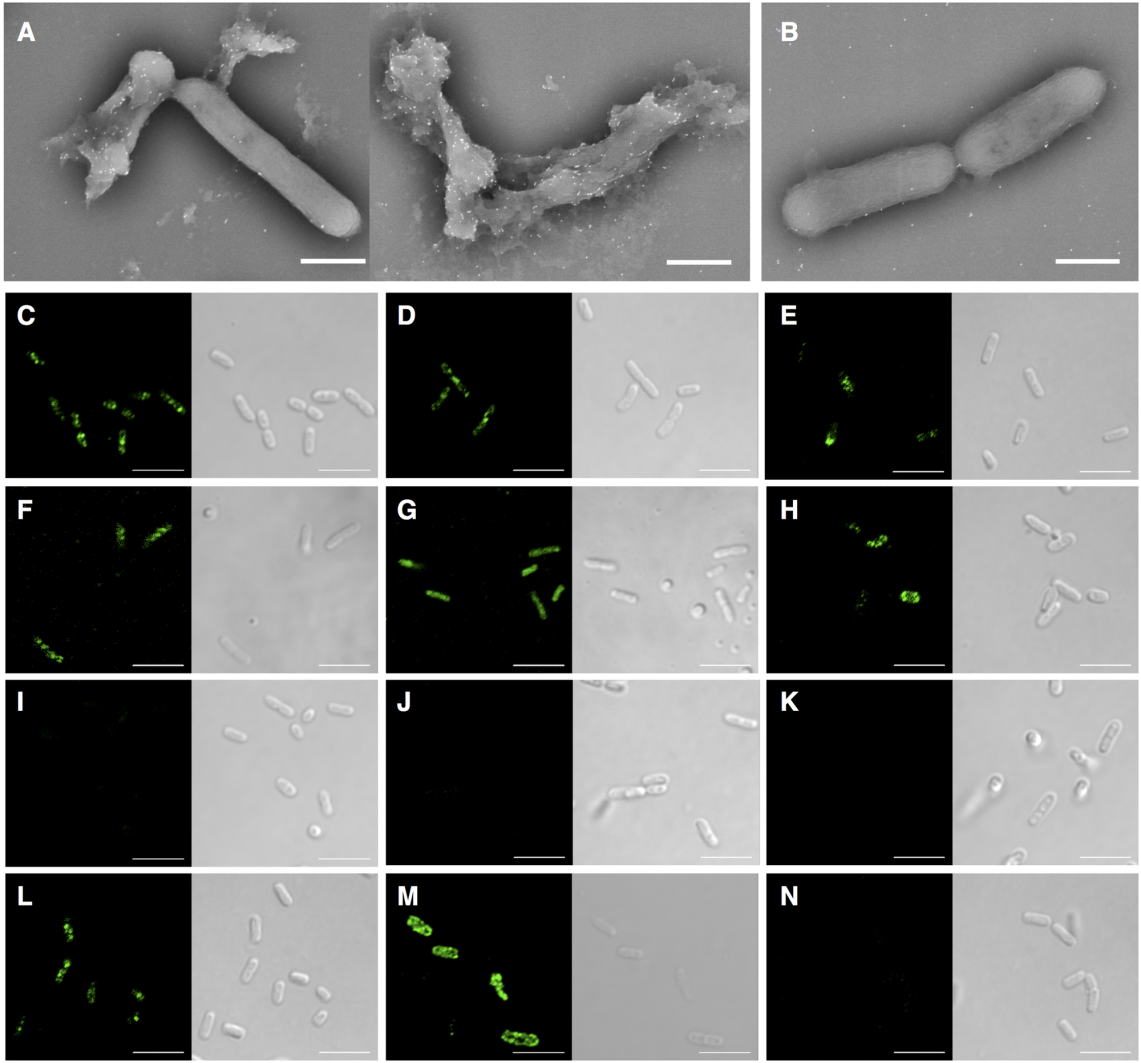
Detection of reactive antibodies. (A) Scanning electron microscopy images of *S*. *sonnei* (HW383) incubated with fresh serum and subsequently reacted with an anti-guinea pig gold-conjugate (white particles). (B) Negative control (unimmunized serum). Scale bars, 1 μm. Immunofluorescence- (*left*) and differential interference contrast- (*right*) based detection of antibodies against (C) *S*. *sonnei* (HW383), (D) *Sd1* (TSH1669), (E) *S*. *flexneri* 1b (9268N), (F) *S*. *flexneri* 3a (GTC-01924) (G) *S*. *flexneri* 6 (GTC-01927), (H) EIEC (NIID1), (I) *Sd1* (MD506), (J) *S*. *flexneri* 1b (9268N17-1) lacking the virulence plasmids, and (K) *S*. *sonnei Δ*T3SS (MS2834). (L) Detection of antibodies against HW383 in sera pre-adsorbed with MS2834. (M) *S*. *flexneri* 2a *ΔinvE* (MF1632) stained the sera used in A to K. (N) Control images of HW383 stained by pre-immune sera. Scale bars, 5 μm.

Experiments were performed at the two facilities. (Fig 1E). For NIID, male Hartley guinea pigs (250–300 g) were purchased from SLC Japan (Fig 2, S2 and S3 Figs), and *S*. *sonnei* (HW383) and *S*. *flexneri* 1b (9268N) were used for the challenge experiments. *Sd1* (TSH1669 and MD506), EIEC (NIID1), and *S*. *flexneri* 1b (9268N and 9268N17-1), 3a (GTC-01924), 6 (GTC-01927) were used for microscopy. For NICED, male non-albino “Old-English-colored” guinea pigs (650–750 g) were used for the colon loop experiments (Fig 1) and for all subsequent experiments (Figs 3–5 and S4 Fig). *Sd1* (NT4907) and *S*. *sonnei* (IDH00968) were used for the challenge experiments.

### Plasmids, mutant strains, and culture conditions

Plasmid pACYC-*ipaBCDA* was constructed by inserting T3SS effector genes (identical to nucleotide sequence 79825–91466; Genbank/EBI Data Bank Accession number CP000039.1) into the *Bam*HI site of pACYC177 in a direction opposite to that of the *tet* promoter [26]. The bacterial strains used are listed in S1 Table. Strains MF1632 and MS2834 were constructed as previously described [27] using the primers listed in S2 Table.

For immunization or challenge, bacterial cells were grown at 37°C in LB Lenox medium as described previously [27], concentrated by centrifugation at 3,000 × g for 5 min at 4°C, and resuspended in PBS. Immunoblotting was performed as described previously [27]. To examine protein expression at 37°C, three independent cultures of 2457T and MF4835 were harvested at the same growth phase (OD_600_ = 0.8), blotted onto the same membrane, and levels of IpaB and InvE were measured using a chemical luminescence-based imaging system (Fusion Solo 7S; VILBER Inc.). Values were calculated relative to those of 2457T (± the standard deviation).

### Invasion assay using HeLa cells

HeLa cells were cultured in 6-well plates at 37°C (5% CO_2_) in Dulbecco’s modified Eagle’s medium (DMEM, Invitrogen) supplemented with 10% fetal bovine serum (FBS). After reaching 60% confluence, Hela cells were washed with sterile PBS and infected with the indicated wild-type and Hfq mutant strains (MOI = 100). Following infection for 30 min, HeLa cells were washed and treated for 1 h with gentamicin (50 μg/ml). Finally, cells were permeabilized with 0.1% Triton X-100, serially diluted, and plated on LB plates prior to CFU counts.

### The colon loop model and bacterial counts in tissues and stools

Six animals were examined in the colon loop model. Three colon segments (each 4 cm in length) per animal were tied with a surgical suture. Then, 1 ml of bacterial suspension in PBS (1.0×10^9^ cfu/segment) or PBS alone was injected directly into the lumen. Inflammation was observed in each of three animals upon sacrifice at 6 or 24 h post-injection. Tissues were fixed and stained with hematoxylin and eosin.

To count the bacteria in the tissues, the intestinal loops were washed twice with PBS containing gentamicin (50 μg/ml), followed by homogenization in 5 ml of PBS. Serial dilutions (10-fold) were prepared and spread on selective agar plates (285310; Hektoen Enteric Agar, Difco). Representative colonies were confirmed in an agglutination test for *S*. *flexneri* 2a (210227; Denka Seiken, Japan). The mean number of cfu/g intestinal tissue was calculated from three animals. For bacterial counts in stool suspensions (1 g stool/ml PBS) were calculated as described above.

### Ocular immunization/ocular challenge

Bacterial strains (5.0×10^8^ cfu/eye) were dropped onto the conjunctival sac of both eyes on two consecutive days; this was repeated 14 days later. At 4 days post-first immunization, the development of keratoconjunctivitis was recorded using a digital camera. Animals were then challenged with *S*. *sonnei* (HW383) and *S*. *flexneri* 1b (9268N). Results were recorded 4 and 3 days after primary inoculation with HW383 and 9268N, respectively. Animals were euthanized at Day 38. Ocular tissues were stained with hematoxylin and eosin (Fig 1E, a).

### Ocular immunization/ocular-intestinal challenge

Immunization was performed using the same dose (5.0×10^8^ cfu/eye) and schedule described in Fig 1E, b, but the left eye was used rather than the right. At Day 28, animals were challenged with the same dose (5.0×10^8^ cfu/eye) of *Sd1* (NT4907), which was placed into the right eye. Keratoconjunctivitis was again observed and recorded at 4 days post-inoculation. On Day 41, animals were fasted for 24 h. On Day 42, the ileocecal junction was inoculated with *S*. *sonnei* (IDH00968) in PBS (1.0×10^9^ cfu/ml) as described previously [33]. Development of watery/bloody diarrhea was monitored for 3 days (Fig 1E, b).

### Oral immunization/intestinal challenge

Two separate experiments were performed for *Sd1* (NT4907) and *S*. *sonnei* (IDH00968). Animals were immunized via the oral route as previously described [34]. Briefly, anesthetized animals were treated with ranitidine (50 mg/kg; intramuscularly), followed by two 5 ml injections (15 min apart) of 5% sodium bicarbonate directly into the stomach via a sterile human infant feeding tube. MF4835 (1.0×10^7^ cfu in 1 ml of PBS), Wild-type strain 2457T (1.0×10^6^ cfu in 1 ml of PBS), or 1 ml of PBS alone was injected through the same tube and animals were returned to the cage. Body weight, rectal temperature, and bacterial counts (cfu) in the stool were measured for 7 days. Immunizations were performed four times, with a 1 week interval between each.

At Day 28, animals were subjected to intestinal challenge with NT4907 or IDH00968 (1.0×10^9^ cfu) as described above. After 24 h, symptoms were recorded and three animals were sacrificed to examine intestinal colonization. The body weight, rectal temperature, and survival of the remaining three animals were recorded for 3 days (Fig 1E, c).

### Measurement of immunoglobulin levels

The levels of IgG and IgA in the serum and sIgA in the stool were measured in ELISA plates coated with strain 2457T, as described previously [33]. Serum/stool samples were serially diluted (2-fold) in PBS (from 1:100 to 1:12,800) and a 100 μl aliquot was tested using HRP-conjugated anti-IgG (A7289; Sigma), -IgA (SA-60-PZ; Immunology Consultant Laboratory Co., USA), or -secretory IgA (KT-55060; Kamiya Biomedical Co., USA) antibodies.

### Detection of serum cytokines by ELISA

TNF-α (E-90113Gu; USCN Co., USA), IFN-γ (E-90049Gu; USCN Co., USA), and IL-6 (MSB701906; MyBioSource Co., USA) were measured using ELISA kits.

### Immunofluorescence microscopy

To eliminate non-specific signals, sera were pre-absorbed with formaldehyde-killed *S*. *sonnei ΔinvE* strain MS1632 and BL21 bacterial cells, which do not express virulence proteins. Cultures were mixed with formaldehyde (final concentration: 1% at OD_600_ = 1.0), fixed for 15 min at 37°C, and the cells washed three times with PBS containing 50 mM glycine. Aliquots (3 μl) of pre-immune and immune serum from the three *Δhfq*-immunized animals were mixed, heated to 56°C for 30 min, diluted (250×) with immunoreaction enhancer solution (NKB-101; Toyobo, Japan) containing 0.02% NaN_3_, and mixed with an excess amounts of killed cells. The pre-absorption (37°C for 1 h) was repeated three times. Absorption by *S*. *sonnei Δ*T3SS strain MS2834 and BL21 was also performed.

To detect reactive antibodies, bacterial strains were harvested at OD_600_ = 0.4, collected by centrifugation at 1,100×g for 5 min at 4°C, suspended in ice-cold TBS (20 mM Tris HCl [pH 7.5], 150 mM NaCl), and attached to poly-L-lysine (P7890; Sigma)-coated cover glasses for 15 min at 4°C. Cover glasses were fixed in paraformaldehyde (4% in PBS) for 15 min, quenched for 5 min with TBS containing 50 mM glycine, and then blocked with 2% BSA in TBS for 15 min followed by TBS containing 5% normal goat serum (pretreated at 56°C for 30 min) for 1 h at 37°C. Cells were washed three times for 5 min with TBS containing 0.05% Tween 20 (TBST). Samples were then incubated with pre-absorbed sera for 1 h at 37°C and washed with TBST. AlexaFluora488-conjugated anti-guinea pig IgG (A11073; Life Technologies) was diluted (100×) with immunoreaction enhancer solution (NKB-101) and incubated with the samples for 1 h at 37°C. The cover glasses were then washed and sealed with 8 μl of Vectashield (Vector labs) and observed under a Carl Zeiss LSM-700 fluorescence microscope (Carl Zeiss LSM-700).

### Electron microscopy

*S*. *sonnei* strain HW383 was harvested at OD_600_ = 0.4, collected by centrifugation at 1,100×g for 5 min at 4°C, suspended in ice-cold TBS, and attached to poly-L-lysine-coated glass plates at 4°C. Cover glasses and attached bacteria were placed in 4-well plates and killed in 0.4 ml of paraformaldehyde (0.5% in PBS) for 15 min, followed by quenching for 5 min in TBS containing 50 mM glycine. Immune serum and serum from unimmunized animals were collected at Day 27 after ocular immunization (Fig 1E, a). Fresh serum was diluted (50×) in TBST, pre-absorbed with the formaldehyde-killed *S*. *sonnei ΔinvE* strain MS1632 and BL21 bacterial cells at 4°C for overnight, and incubated with the glass plates for 1 h at 37°C. After washing with PBST, the plates were blocked with 2% BSA and 5% heat-treated goat serum, washed again with PBST, overlaid with a 15 nm gold-conjugated anti-guinea pig antibody (815.144; Aurion Immuno Gold, 100× dilution in NKB-101), and incubated at 37°C for 1 h. After washing, the plates were fixed with 2.5% glutaraldehyde/2% paraformaldehyde and observed under a Hitachi SU6600 scanning electron microscope. Although nearly 5% of cells in samples treated with immunized serum were destroyed, all cells in samples treated with pre-immune serum were intact.

## Results

### Characterization and selection of the vaccine candidate

Consistent with our previous studies [27–29], we found that a mutant of *S*. *flexneri* 2a harboring a deletion of the *hfq* gene (*Δhfq*: MF4835) expressed InvE and a T3SS effector (IpaB) at the repressive temperature of 30°C (S1A Fig). At 37°C, expression was significantly higher than that in the wild-type strain (Wt: 2457T) [InvE: × 1.67±0.29, *p*<0.05, IpaB: × 1.49±0.17, *p*<0.05 (n = 3)], leading to a significantly higher rate of invasion into cultured cell lines (S1B Fig). Also, when 1.0×10^9^ cfu of bacteria were injected into colon loop segments *in vivo*, the mutant caused less severe symptoms (Fig 1A, a and 1B); also, a high invasion rate was observed at the early stage (6 hr) (Fig 1D, white bars). By contrast, the Wt strain induced bleeding and tissue distraction (Fig 1A, c and 1C).

At first, we assumed that effectors of T3SS, such as IpaBCDA, would be the most important antigens; therefore, we transformed the *Δhfq* mutant with a multi-copy plasmid encoding *ipaBCDA* genes to increase expression of such antigens (S1A Fig). The strain carrying the *ipaBCDA* plasmid (MF4837) and the *Δhfq* and Wt strains were tested by ocular vaccination into Hartley guinea pigs [35]. Bacteria (5.0×10^8^ cfu / eye) were dropped into both eyes on two consecutive days; this procedure was repeated 2 weeks later (Fig 1E, a).

Animals immunized with *Δhfq* (Fig 2A) or *Δhfq* harboring the *ipaBCDA* plasmid (Fig 2B) developed keratoconjunctivitis, which was milder than that observed in animals immunized with the Wt strain (Fig 2C). The eyes of all animals recovered by the time of the second immunization, which did not cause a second infection. On Day 28 post-first immunization, animals were challenged in both eyes with the same dose of *S*. *sonnei* (HW383). Unexpectedly, all three animals immunized with the *Δhfq* strain remained asymptomatic throughout the observation period (Fig 2E, S2 Fig). The five animals immunized with *Δhfq* carrying the *ipaBCDA-*encoding plasmid (Fig 2F), as well as all Wt-immunized animals (Fig 2G), developed opaque corneas, although this was more pronounced in the Wt-immunized group. All three animals treated with PBS showed damage to the corneal surface, which was accompanied by excretion of pus (Fig 2H).

After recovering from the first challenge with *S*. *sonnei*, animals were challenged with *S*. *flexneri* 1b strain 9268N (5.0×10^8^ cfu/eye) on Day 35. Two animals in the *Δhfq-*immunized group were asymptomatic (S3 Fig). The remaining animal showed increased opacity of the left cornea only, but recovered within a few days. Animals immunized with the Wt strain or with *Δhfq* carrying the *ipaBCDA* plasmid again developed opaque corneas, although three animals appeared asymptomatic throughout the observation period (S3 Fig).

Microscopic examination of the cornea at Day 38 indicated an intact structure in *Δhfq*-immunized animals (Fig 2I) and loss of normal structure in PBS-treated animals (Fig 2L). Animals immunized with the *Δhfq* strain carrying the *ipaBCDA-*encoding plasmid (Fig 2J) and those immunized with the Wt strain (Fig 2K) showed vesicular degeneration and thinning of the eosinophilic layer, both of which were more evident in the Wt-immunized group. The group immunized with bacteria harboring the *ipaBCDA*-encoding plasmid showed adverse effects, possibly due to changes in optimal T3SS expression due to the multiple *ipa* genes encoded by the plasmid. Therefore, we used the plasmid-negative *Δhfq* strain in all subsequent experiments performed at the NICED (where we used a non-albino breed of guinea pig).

### Induction of systemic immunity by ocular inoculation

We next examined whether vaccination protected non-albino guinea pigs against *Sd1*. Animals were immunized in the left eye (5.0×10^8^ cfu) on Days 0, 1, 14, and 15, and then subsequently challenged (on Day 28) by administration of the same dose into the right eye (Fig 1E, b). No bacteria were transmitted from the left to the right eye. Animals immunized with the Wt strain (2457T) developed severe keratoconjunctivitis (Fig 3B; one animal died from systemic infection and three remained infected beyond Day 28 (S4 Fig)); however, animals immunized with *Δhfq* (MF4835) appeared asymptomatic (Fig 3A). Since Hartley guinea pigs developed distinct lesions after immunization with the same *Δhfq* strain (Fig 2A), it appears that different animal breeds show differing susceptibility to infection.

On Day 28, the right eye of each animal was challenged with the *Sd1* strain (NT4907). The *Δhfq*-immunized group was again protected against subsequent *Sd1* challenge (Fig 3D), which induced keratoconjunctivitis in all PBS-treated animals (Fig 3F). The Wt-immunized group was also asymptomatic (Fig 3E). The finding that Wt-immunized Hartley guinea pigs developed minor symptoms (Fig 2G) again highlights potential differences in susceptibility between the two guinea pig breeds.

After recovering from the *Sd1* infection (Day 42), animals were subjected to intestinal challenge with *S*. *sonnei* strain (IDH00968) via direct inoculation into the colon with concomitant ligation of the cecum to promote infection [33]. PBS-treated animals developed frequent and bloody diarrhea, and all died within 2 days (Fig 3G). By contrast, 2/6 animals in the *Δhfq*-immunized group and 2/5 in the Wt-immunized group developed mild watery diarrhea, but all recovered within a few days. The remaining animals were asymptomatic.

### Oral immunization and intestinal challenge

The above results indicate that ocular immunization with *Δhfq* elicited a systemic immune response against *Shigella* strains of heterologous serotype. Thus, we next examined an oral immunization model of shigellosis, which was established in achlorhydric animals treated with H_2_ blockers [34]. Two independent experiments using *Sd1* and *S*. *sonnei* (see S1 Text) were conducted. Animals were immunized with 1.0×10^7^ cfu *Δhfq* (MF4835)/week or with 1.0×10^6^ cfu Wt (2457T)/week for 4 weeks (Fig 1E, c). The dose of the Wt strain was reduced to 1.0 × 10^6^ cfu due to observed lethality when used at 1.0×10^7^ cfu in the preliminary experiment (see Discussion section). At 1 day post-first immunization, the *Δhfq-*immunized group showed an increase in rectal temperature (Fig 4A) [*p*<0.01] and weight loss (Fig 4B) [*p*<0.05] without diarrhea; this was not true for the PBS-treated group. The Wt-immunized group developed watery diarrhea, an increased rectal temperature, and weight loss (maximum 11% at Day 3 when compared with the average for PBS-treated animals). Although *Δhfq*-immunized animal received more bacteria than Wt-immunized animal, fewer were isolated from stool samples on Day 1 [*p*<0.01]; also, the *Δhfq*-immunized animals cleared bacteria faster than the Wt-immunized group (Fig 4C). After recovering from the first immunization, none of the *Δhfq-*, Wt-, or PBS-treated animals developed diarrhea after subsequent immunization, suggesting induction of a protective immune response against *Shigella* strains of the same serotype.

Although production of serum IgG (Fig 4D) by animals in the *Δhfq*-immunized group was delayed slightly, the levels at Day 28 were the same as those in the Wt-immunized group. The levels of IgA in the serum (Fig 4E) and of secreted IgA (Fig 4F) in the stools of the *Δhfq-*immunized group were similar to those in the Wt-immunized group at Day 28 [*p* = 0.18]. We then compared cytokine levels on Days 7 and 28. In line with the severity of infection, the levels of TNF-α, IL-6, and IFN-γ were significantly higher in the Wt-immunized group than in the *Δhfq-*immunized group (Fig 4G, 4H, and 4I).

Immunized animals were subjected to intestinal challenge with *Sd1* at Day 28 post-first immunization. All PBS-treated animals excreted frequent watery stools, and 50% developed bloody diarrhea. However, 4/6 animals in both the *Δhfq*- and Wt-immunized groups were asymptomatic. The remaining animals excreted mucoidal stools, and 50% showed a small amount of bleeding at 24 h post-challenge. At this point, the bacterial counts in intestinal tissues from both immunized groups were significantly lower than those in the PBS controls. However, there was no significant difference in bacterial counts between *Δhfq-* and Wt-immunized animals (Fig 4J). Data regarding rectal temperature (Fig 4K), body weight (Fig 4L), and survival (Fig 4M) supported the generation of a protective immune response in the immunized groups. Microscopic observation of intestinal tissues after *Sd1* challenge revealed no evidence of bleeding, although a limited number of erythrocytes was observed in the intestinal lumen of PBS-treated animals. Loss of microvilli and invasion of polymorphonuclear leukocytes into the lamina propria were also observed in PBS-treated animals; tissues from Wt- and *Δhfq*-immunized animals appeared normal (Fig 4N).

### Detection of antibodies common to *Shigella* strains

The results of the above experiments indicated that the *Δhfq* strain induced production of protective antibodies against heterologous serotypes of *Shigella*. Consistent with this, *S*. *sonnei* cells were lysed by fresh serum from *Δhfq*-immunized animals (Fig 5A) but not by serum from pre-immune animals (Fig 5B), suggesting complement activation by specific antibodies. Serum samples pre-absorbed with *S*. *sonnei ΔinvE* and *E*. *coli* BL21 cells reacted with Wt *S*. *sonnei* (Fig 5C), *Sd1* (Fig 5D), *S*. *flexneri* strains of serotype 1b (Fig 3E), 3a (Fig 5F), and 6 (Fig 5G), and with enteroinvasive *E*. *coli* (EIEC) carrying a virulence plasmid [9] similar to that harbored by *Shigella* (Fig 5H). Sera did not react with an avirulent *Sd1* strain (Fig 5I) or with *S*. *flexneri* 1b (Fig 5J) lacking virulence plasmids, indicating that antibodies are specific for a virulence factor(s) encoded by the virulence plasmids. A deletion mutant of *S*. *sonnei Δ*T3SS (MS2834 *ΔipaA~spa40*) was not stained by the sera (Fig 5K), whereas sera pre-absorbed with the *Δ*T3SS strain and BL21 reacted with Wt *S*. *sonnei* (Fig 5L). This indicates that these antibodies at least recognize proteins within the T3SS-encoding region. Finally, *S*. *flexneri* 2a *ΔinvE* strain, which expresses serotype antigens but not virulence proteins (S1A Fig), reacted strongly with serotype-specific antibodies produced upon subsequent immunization with *S*. *flexneri* 2a *Δhfq* (Fig 5M). No signal was detected in Wt *S*. *sonnei* reacted with serum from pre-immune animals (Fig 5N).

## Discussion

In the present study, animals immunized with a *S*. *flexneri* 2a-based *Δhfq* strain were protected from heterologous challenge with *Sd1* and *S*. *sonnei*. The results provide strong evidence supporting cross protection against *Shigella* strains of heterologous serotypes; these results were replicated in independent guinea pig models.

The amount of bacteria (5.0×10^8^ cfu for ocular immunization and 1.0×10^6^–10^7^ cfu for oral immunization) was much higher than that usually required to cause diarrhea in humans (1×10^2^ ~10^3^ cfu) [36], leading us to postulate that cross-protection was induced by administration of *Shigella* strains at excess amounts. Exposure to a sufficient number of bacteria expressing common virulence proteins could induce immunity and broad protection, which may not be fully established during a natural infection cycle during which limited bacteria begin to propagate within intracellular spaces within the colon epithelium, thereby escaping from the host immune system. Indeed, several early studies of a keratoconjunctivitis model documented cross protection, albeit partial, which support this hypothesis. Serény (who first established this animal model) and other groups reproducibly documented that keratoconjunctivitis induced partial protection against reinfection of the same eye by *Shigella* strains of heterologous serotypes, although the other eye was susceptible [37–39]. Also, Adamus *et al*. report partial and complete induction of systemic immunity in guinea pigs and rabbits, respectively, after subcutaneous immunization with outer membrane proteins from *S*. *sonnei*, resulting in protection from subsequent ocular challenge with *S*. *flexneri* 3a [40].

These data provide possible clues to understanding the results of studies using strain T_32_–Istrati [20, 22], which harbors three deletions (*ipaBCD*, *invA* [corresponding to the region around *spa32*] and *virG*) in the virulence plasmid [21]. If expression of the T3SS transporter complex (encoded by *mxi-spa* region) itself remains intact, administration of a high dose might support immunization by common virulence proteins.

Strain T_32_-Istrati was later modified by transformation with a virulence plasmid from *S*. *sonnei* harboring *rfb* (to drive O-antigen biosynthesis) and two deletions (*ΔvirF* and a 37 kbp segment encoding the whole of T3SS {*ΔinvE~ipaBCDA~mxi~spa40*}), leading to production of O-antigens from both *S*. *flexneri* 2a and *S*. *sonnei*. Field trials showed protection against *S*. *flexneri* 2a (61.07%) and *S*. *sonnei* (72.48%). A review of the literature suggests that it provided 41.89% protection against other serotypes [41], indicating that it is less effective than the original studies suggest. In the context of our hypothesis that cross protection is achieved by immunization with common virulence proteins, the low cross protection efficacy could be attributed to a lack of virulence gene expression after deletion of T3SS and the essential regulator *virF*, which results in loss of immunogenicity induced by common virulence proteins.

A non-human primate (NHP) study using 2457T does not support previous data showing cross protection [16]. We have no explanation for this; however, large amounts of bacteria (1×10^10^ cfu) are generally required to induce onset of diarrhea. Therefore, some researchers consider NHP models unsuitable for evaluation of vaccines [3]. Another possible (but less likely) explanation could be differences in the bacterial strains, which were distributed to each laboratory a long time ago. Also, claims that protection was specific to guinea pigs may be based on the fact that successful immunization was achieved by using excess amounts of bacteria. However, these questions require clarification in further studies enrolling human volunteers and using attenuated strains. The colon loop model demonstrated effective attenuation of the *Δhfq* strain without loss of expression of virulence genes (Fig 1B). Inoculation of the colon loop with excess amounts of bacteria (1.0×10^9^ cfu) resulted in a greater number of locally-invading bacteria (Fig 1D) without any symptoms, indicating that attenuation afforded by the *Δhfq* mutation was so effective that fewer side effects emerged, irrespective of the inoculation dose. This is a great advantage in terms of practical use.

Experiments using two different breeds of guinea pig highlighted different responses against infection, although the use of two models arose because of difficulties with international transfer of materials. Hartley guinea pigs developed corneal lesions after inoculation with the *Δhfq* mutant (Fig 2A), whereas non-albino guinea pigs were asymptomatic (Fig 3A). After challenge, Hartley guinea pigs immunized with the Wt strain showed opaque changes in the cornea (Fig 2G), which were not evident in the experiment involving non-albino guinea pigs (Fig 3E). Different breeds of guinea pig show differing susceptibility to infection by pathogens [42]. Also, the difference might be due to the composition of intestinal flora. Hartley guinea pigs were purchased from a commercial farm on which animals were kept under strict sanitary conditions (including air and diet). Non-albino animals were bread at NICED, and so the same level of sanitary control was not possible. Such differences would affect immunity against infection by microorganisms, and possibly by the highly-attenuated *Shigella* strain.

Rodent models of oral infection by *Shigella* are under development. Here, we developed a new guinea pig model based on achlorhydric treatment; this approach was first used to develop a rabbit model of enterohemorrhagic *E*. *coli* infection [34]. Preliminary experiments conducted with 1.0×10^7^ cfu of both the Wt and *Δhfq* strains revealed a high efficacy of infection, resulting in the death of all six Wt-immunized animals. As a challenge that induces lethal damage, an excess amount of bacteria (1.0×10^9^ cfu) was directly inoculated into the colon of all animals. Since the body weight of the two immunized groups remained constant, or even increased, at Day 28–30, the surgical procedure used for the inoculation had a minimal effect on the condition of the animals.

Expression of IFN-γ and its receptor increases in patients with shigellosis, and further increases during the convalescence period [43]. The IFN-γ and immunoglobulin responses in animals immunized with the *Δhfq* strain induced immunity comparable with that induced by the Wt strain. In the two independent experiments, the body weight of all Wt-immunized animals was at least 5% higher than that of the other groups at Day 28, reflecting the reproducibility of the two experiments. This increase may be due to the stress of severe diarrhea, which might encourage excess uptake of food.

Immunological detection of induced antibodies was consistent with the protective effects observed in the challenge studies. Also, detection of antibodies against different strains, including EIEC, suggests positive responses against universal serotypes of *Shigella* strains. We did not conduct challenge experiments using *S*. *flexneri* 2a (which has a serotype identical to that of the immunizing strain) because the majority of studies, including one using the *S*. *typhimurium Δhfq* vaccine [32], report generation of immunity against strains of homologous serotype. Production of antibodies against homologous O-antigen was detected using the *ΔinvE* mutant of *S*. *flexneri* 2a strain 2457T (which has lost expression of all virulence proteins), as evidenced by the strongest signal upon immune analysis. Consistent with this, sera from immunized animals strongly agglutinated 2457T cells, but not *S*. *sonnei* or *Sd1* cells. In addition, *Shigella*-specific proteins lacking InvE-dependent regulation could be considered potential antigens. However, if these proteins were common among *Shigella* species, detection of antibodies would be difficult in the experiment that required absorption of serum with the *ΔinvE* strain of *S*. *sonnei* to reduce non-specific signals generated by general bacterial proteins.

A rough estimate suggests that vaccines that are effective against both serotypes of *S*. *sonnei* and *S*. *flexneri* 2a, 3a, 6 will cover about 60% of patients [7, 44]. Detection of antibodies against these serotypes indicates a potentially broad effect for prevalent strains. In addition, the O-antigen of *S*. *flexneri* 2a provides cross protection against *S*. *flexneri* strains 1a, 2b, 3b, 4a, 5a, and Y, all of which possess group factor 3, 4 and type factor II [45]. This provides a *S*. *flexneri* 2a-based vaccine with a great advantage over other vaccine candidates that target a limited number of virulence proteins.

Immunization with common virulence proteins from an attenuated mutant is a new concept; fortunately, a single mutation attenuated the immunizing strain and increased antigen expression. These results suggest that such a strategy could be applied to other pathogens harboring common virulence machinery if one carefully selects the appropriate strain and mutation to provide effective attenuation without loss of antigen expression. Common virulence protein antigens expressed by the attenuated strain and acting as “live toxoids” are expected to elicit the same levels of host immunity against multiple serotypes of pathogen as that elicited by conventional toxoids.

## Supporting information

S1 Text Oral immunization and intestinal challenge with *S*. *sonnei* strain IDH00968. Oral immunization and intestinal challenge with *S*. *sonnei* strain IDH00968 was also performed according to the schedule shown in Fig 1E, c using the same doses of bacteria (1.0×10^7^ cfu for the *Δhfq* strain and 1.0×10^6^ cfu for the Wt strain) used for *Sd1* challenge. One day after the first immunization, the *Δhfq-*immunized group showed a significant increase in rectal temperature [*p*<0.05] and significant loss of body weight [*p*<0.01] (without diarrhea) when compared with the PBS-treated group (S5A and S5B Fig); these findings are similar to those after initial *Sd1* challenge (Fig 4A and 4B). Also, immunoglobulin and cytokine levels (S5C–S5H Fig) were similar to those measured after *Sd1* challenge (Fig 4D–4I). After intestinal challenge at Day 28, all six PBS-treated animals developed frequent watery diarrhea, whereas four of the six subsequently developed bloody diarrhea. However, 4/6 animals in the *Δhfq*-immunized group and 5/6 animals in the Wt-immunized group were asymptomatic. The remaining animals excreted mucoidal stools, and all showed small amount of bleeding at 24 h post-challenge. Bacterial colonization of intestinal tissues at this point was significantly lower than that in PBS controls, with no significant difference between *Δhfq-* and Wt-immunized animals (S5I Fig). Infection with *S*. *sonnei* strain IDH00968 appeared more severe than infection with *Sd1*. PBS-treated animals showed an increase in body temperature, loss of body weight, and reduced survival (S5J–S5L Fig). Observation of tissues excised from PBS-treated animals revealed bleeding and tissue destruction. Hyperplastic goblet cells and a normal epithelial structure were observed in both Wt- and *Δhfq*-immunized animals (S5M Fig).
(DOCX)

S1 Fig Expression of the IpaB and InvE proteins by *S*. *flexneri* 2a strains and the results of the invasion assay. (A) Wt (2457T), lanes *1* and *6*; *hfq* (MF4835), lanes *2* and *7*; two transformants of *hfq* carrying the pACYC-*ipaBCDA* plasmid (MF4837), lanes *3* and *8* and lanes *4* and *9*; and *ΔinvE* (MF1632), lanes *5* and *10*. Each lane contains 5 μl of whole culture. The antibodies used for detection are indicated on the *left*. Hns was used as the loading control. (B) Invasion of HeLa cells. White and gray bars denote *Δhfq* and Wt strains, respectively. Values are expressed as the mean ± SD; n = 3. **p<0.01.
(TIFF)

S2 Fig Eyes of all animals photographed 4 days post-challenge with *S*. *sonnei* strain HW383. Animals were immunized with *Δhfq* (MF4835), *Δhfq* carrying the *ipaBCDA* plasmid (MF4837), or the Wt strain (2457T). Animals showing no symptoms are denoted by an *asterisk*.
(TIFF)

S3 Fig Eyes of all animals photographed 3 days after challenge with *S*. *flexneri* 1b strain 9268N. Animals were immunized with *Δhfq* (MF4835), *Δhfq* carrying the *ipaBCDA* plasmid (MF4837), or Wt (2457T). Animals with no symptoms are denoted by an *asterisk*.
(TIFF)

S4 Fig Photographs showing the eyes of all animals at 4 days post-challenge with *Sd1* strain NT4907 (right eye). Animals were immunized with *Δhfq* (MF4835) or Wt (2457T).
(TIFF)

S5 Fig Oral immunization and subsequent intestinal challenge with *S*. *sonnei* strain IDH00698. Changes in (A) rectal temperature and (B) body weight. Levels of (C) IgG and (D) IgA in serum samples, and of (E) secretory IgA in stool samples. Levels of (F) TNF-α, (G) IL-6, and (H) IFN-γ in serum. Gray and white bars indicate values at Days 7 and 28, respectively. Symbols: blue square, *Δhfq*; orange diamond, Wt; black triangle, PBS. Values are expressed as the mean ± SD; n = 6. *p<0.05; **p<0.01; n.s., not significant. (I) Intestinal colonization in three animals at 24 h post-*S*. *sonnei* challenge. Changes in (J) rectal temperature and (K) body weight. Symbols: blue square, *Δhfq*; orange diamond, Wt; black triangle, PBS. Values are expressed as the mean ± SD; n = 3. Values derived from fewer than three animals are indicated by a dashed line. (L) Survival curves. (M) Microscopic observation of tissues from animals immunized with *Δhfq* (a), Wt strain (b), or PBS (c). Scale bars, 100 μm.
(TIFF)

S1 Table Bacterial strains used in the study. (DOCX)

S2 Table Primers used in the study. (DOCX)
