# Supplementary material for: An attenuated Shigella mutant lacking the RNA-binding protein Hfq provides cross-protection against Shigella strains of broad serotype
Source: PLoS Negl Trop Dis. 2017 Jul 20;11(7):e0005728. doi: 10.1371/journal.pntd.0005728 (PMC5544247; doi:10.1371/journal.pntd.0005728)
Supplement: S1 Text — Oral immunization and intestinal challenge with S. sonnei strain IDH00968 was also performed according to the schedule shown in Fig 1E, c using the same doses of bacteria (1.0×10^7^ cfu for the Δhfq strain and 1.0×10^6^ cfu for the Wt strain) used for Sd1 challenge. One day after the first immunization, the Δhfq-immunized group showed a significant increase in rectal temperature [p<0.05] and significant loss of body weight [p<0.01] (without diarrhea) when compared with the PBS-treated group (S5A and S5B Fig); these findings are similar to those after initial Sd1 challenge (Fig 4A and 4B). Also, immunoglobulin and cytokine levels (S5C–S5H Fig) were similar to those measured after Sd1 challenge (Fig 4D–4I). After intestinal challenge at Day 28, all six PBS-treated animals developed frequent watery diarrhea, whereas four of the six subsequently developed bloody diarrhea. However, 4/6 animals in the Δhfq-immunized group and 5/6 animals in the Wt-immunized group were asymptomatic. The remaining animals excreted mucoidal stools, and all showed small amount of bleeding at 24 h post-challenge. Bacterial colonization of intestinal tissues at this point was significantly lower than that in PBS controls, with no significant difference between Δhfq- and Wt-immunized animals (S5I Fig). Infection with S. sonnei strain IDH00968 appeared more severe than infection with Sd1. PBS-treated animals showed an increase in body temperature, loss of body weight, and reduced survival (S5J–S5L Fig). Observation of tissues excised from PBS-treated animals revealed bleeding and tissue destruction. Hyperplastic goblet cells and a normal epithelial structure were observed in both Wt- and Δhfq-immunized animals (S5M Fig). (DOCX) [file pntd.0005728.s001.docx]

**S1 Text. Oral immunization and intestinal challenge with *S. sonnei* strain IDH00968.**

Oral immunization and intestinal challenge with *S. sonnei* strain IDH00968 was also performed according to the schedule shown in Figure 1E, c using the same doses of bacteria (1.0×10^7^ cfu for the *Δhfq* strain and 1.0×10^6^ cfu for the Wt strain) used for *Sd1* challenge. One day after the first immunization, the *Δhfq-*immunized group showed a significant increase in rectal temperature [*p*<0.05] and significant loss of body weight [*p*<0.01] (without diarrhea) when compared with the PBS-treated group (S5 Fig. A and B); these findings are similar to those after initial *Sd1* challenge (Figs. 4A and 4B). Also, immunoglobulin and cytokine levels (S5 Fig. C to H) were similar to those measured after *Sd1* challenge (Figs. 4D–I). After intestinal challenge at Day 28, all six PBS-treated animals developed frequent watery diarrhea, whereas four of the six subsequently developed bloody diarrhea. However, 4/6 animals in the *Δhfq*-immunized group and 5/6 animals in the Wt-immunized group were asymptomatic. The remaining animals excreted mucoidal stools, and all showed small amount of bleeding at 24 h post-challenge. Bacterial colonization of intestinal tissues at this point was significantly lower than that in PBS controls, with no significant difference between *Δhfq-* and Wt-immunized animals (S5 Fig. I). Infection with *S. sonnei* strain IDH00968 appeared more severe than infection with *Sd1*. PBS-treated animals showed an increase in body temperature, loss of body weight, and reduced survival (S5 Fig. J–L). Observation of tissues excised from PBS-treated animals revealed bleeding and tissue destruction. Hyperplastic goblet cells and a normal epithelial structure were observed in both Wt- and *Δhfq*-immunized animals (S5 Fig. M).
