# Supplementary figures and images for: An attenuated Shigella mutant lacking the RNA-binding protein Hfq provides cross-protection against Shigella strains of broad serotype
Source: PLoS Negl Trop Dis. 2017 Jul 20;11(7):e0005728. doi: 10.1371/journal.pntd.0005728 (PMC5544247; doi:10.1371/journal.pntd.0005728)

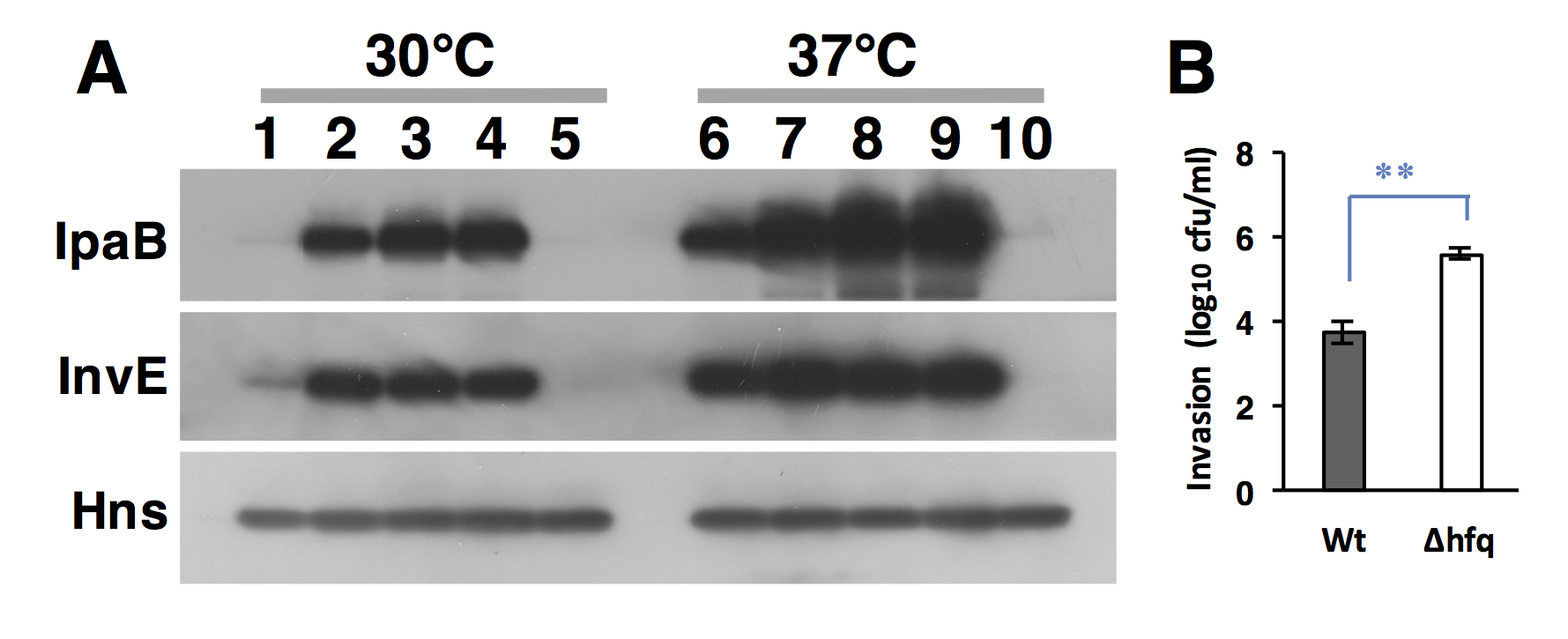

Supplement: S1 Fig — (A) Wt (2457T), lanes 1 and 6; hfq (MF4835), lanes 2 and 7; two transformants of hfq carrying the pACYC-ipaBCDA plasmid (MF4837), lanes 3 and 8 and lanes 4 and 9; and ΔinvE (MF1632), lanes 5 and 10. Each lane contains 5 μl of whole culture. The antibodies used for detection are indicated on the left. Hns was used as the loading control. (B) Invasion of HeLa cells. White and gray bars denote Δhfq and Wt strains, respectively. Values are expressed as the mean ± SD; n = 3. **p<0.01. (TIFF) [file pntd.0005728.s002.tiff]

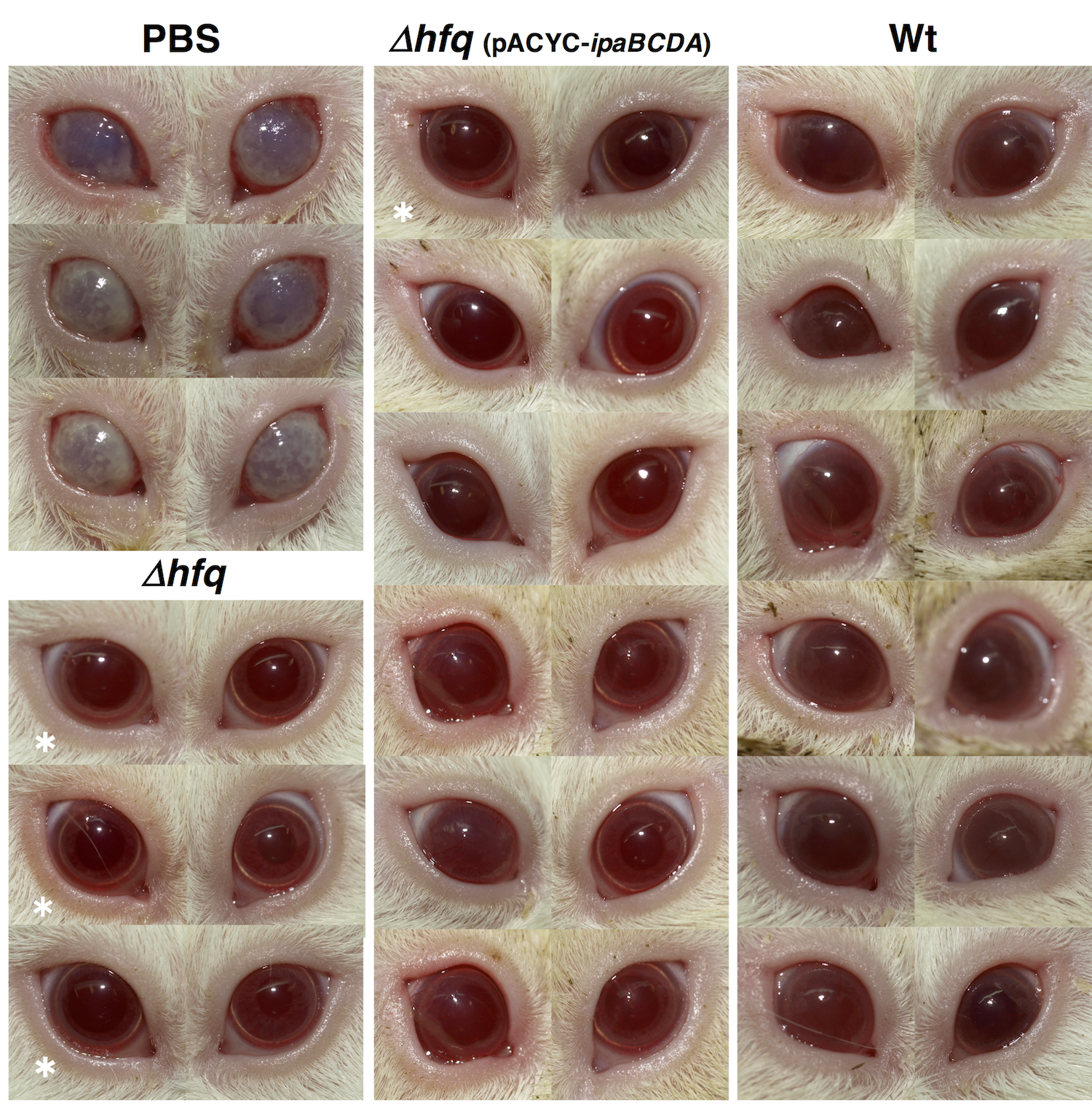

Supplement: S2 Fig — Animals were immunized with Δhfq (MF4835), Δhfq carrying the ipaBCDA plasmid (MF4837), or the Wt strain (2457T). Animals showing no symptoms are denoted by an asterisk. (TIFF) [file pntd.0005728.s003.tiff]

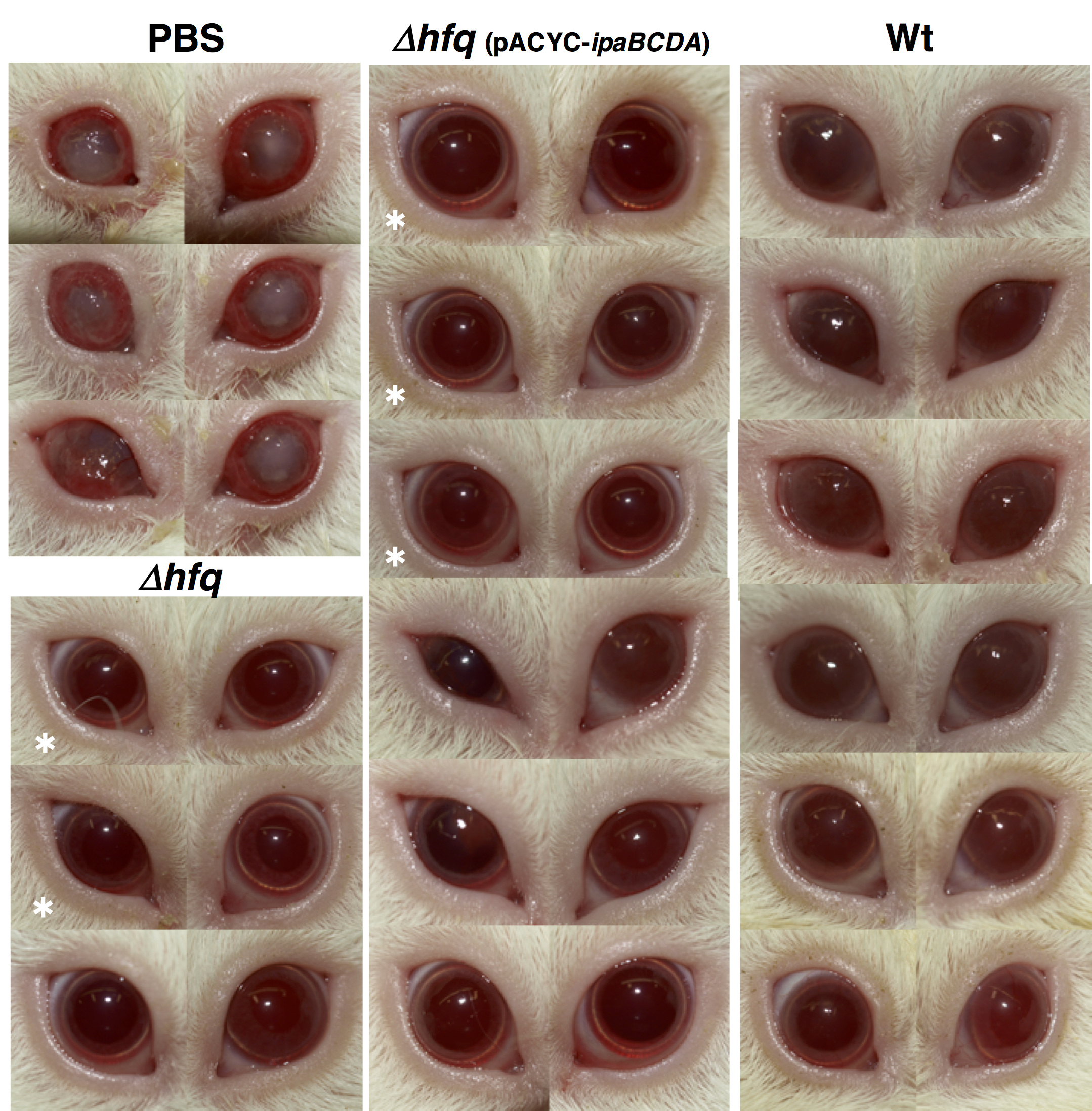

Supplement: S3 Fig — Animals were immunized with Δhfq (MF4835), Δhfq carrying the ipaBCDA plasmid (MF4837), or Wt (2457T). Animals with no symptoms are denoted by an asterisk. (TIFF) [file pntd.0005728.s004.tiff]

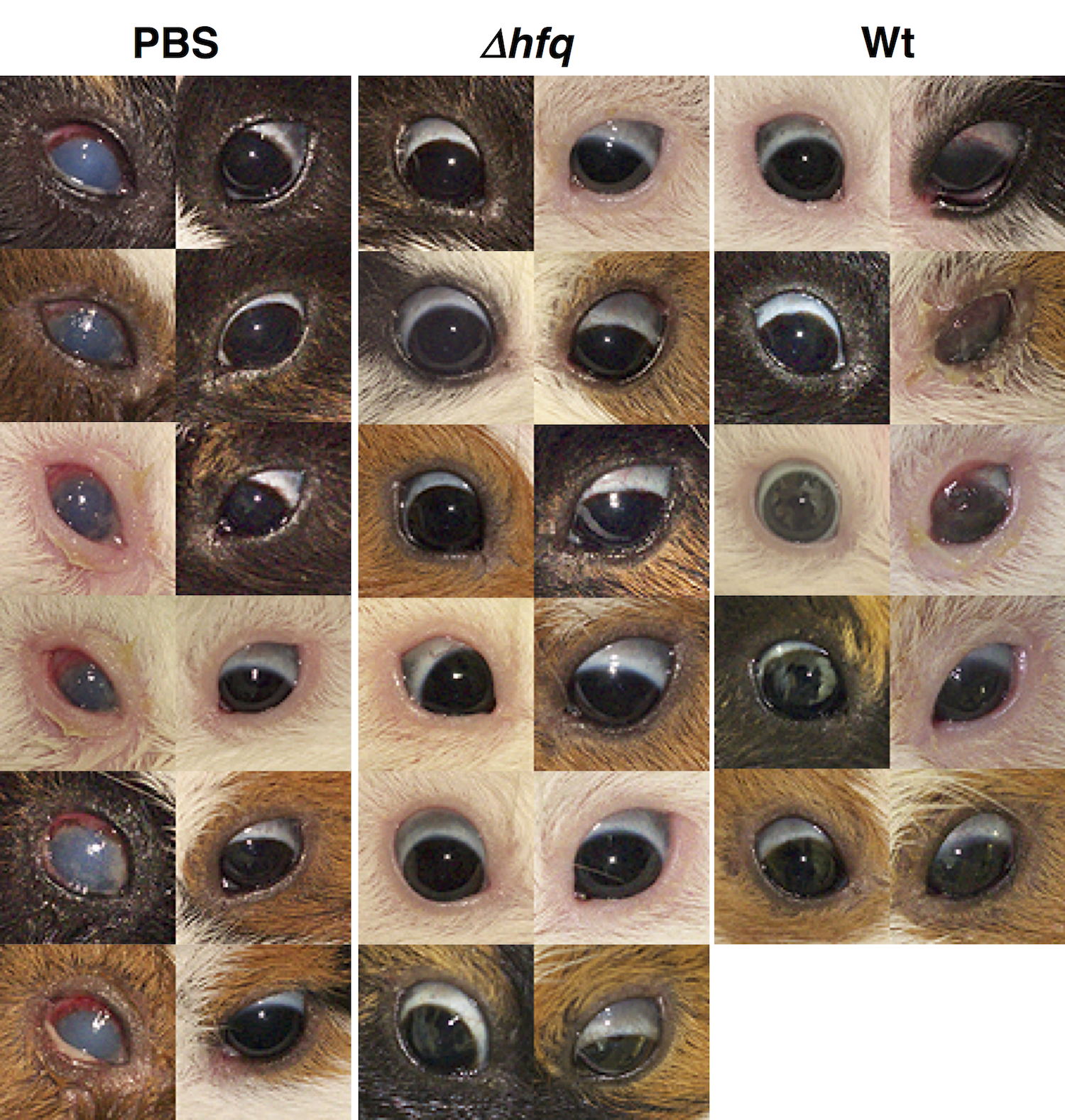

Supplement: S4 Fig — Animals were immunized with Δhfq (MF4835) or Wt (2457T). (TIFF) [file pntd.0005728.s005.tiff]

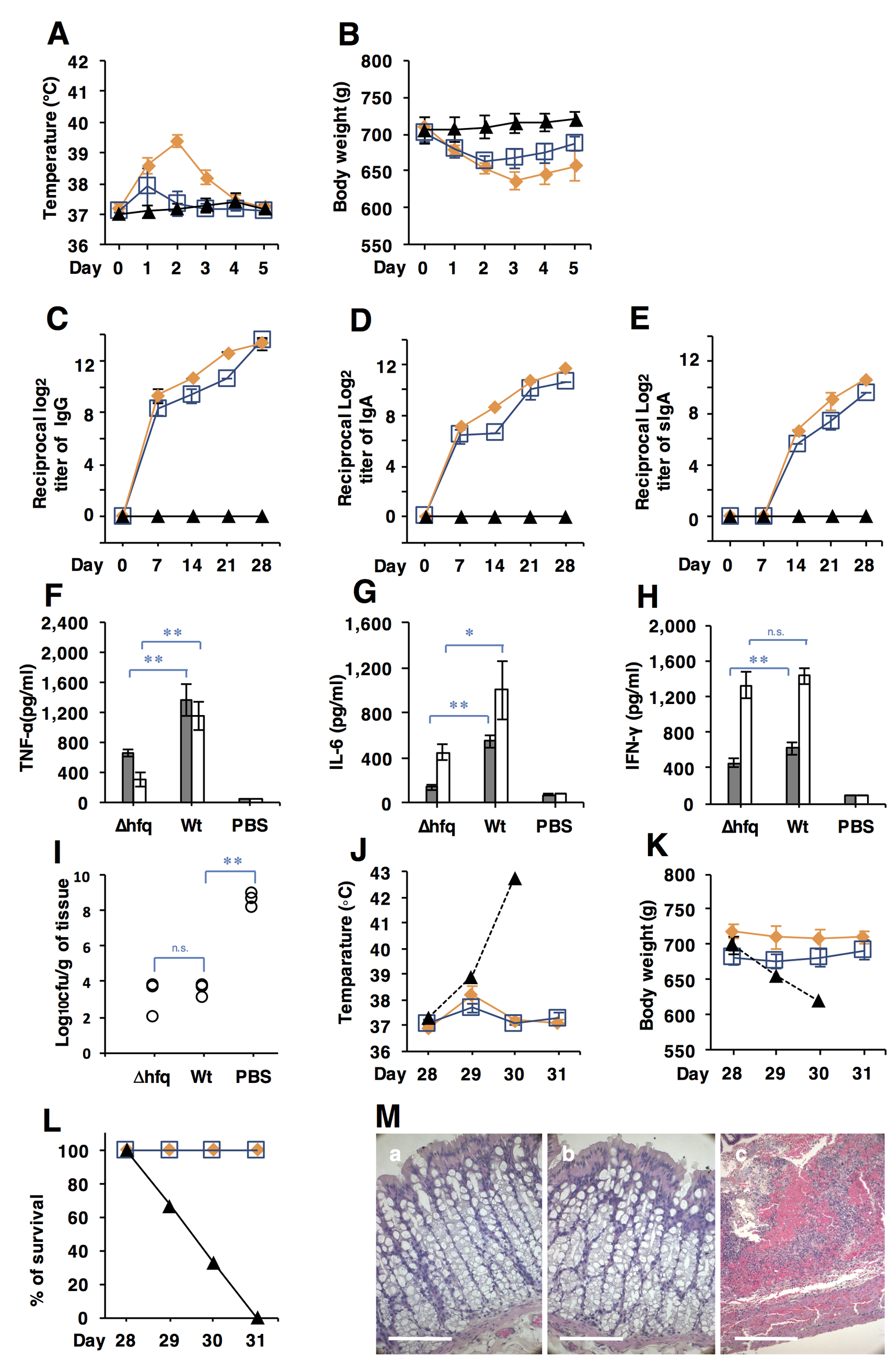

Supplement: S5 Fig — Changes in (A) rectal temperature and (B) body weight. Levels of (C) IgG and (D) IgA in serum samples, and of (E) secretory IgA in stool samples. Levels of (F) TNF-α, (G) IL-6, and (H) IFN-γ in serum. Gray and white bars indicate values at Days 7 and 28, respectively. Symbols: blue square, Δhfq; orange diamond, Wt; black triangle, PBS. Values are expressed as the mean ± SD; n = 6. *p<0.05; **p<0.01; n.s., not significant. (I) Intestinal colonization in three animals at 24 h post-S. sonnei challenge. Changes in (J) rectal temperature and (K) body weight. Symbols: blue square, Δhfq; orange diamond, Wt; black triangle, PBS. Values are expressed as the mean ± SD; n = 3. Values derived from fewer than three animals are indicated by a dashed line. (L) Survival curves. (M) Microscopic observation of tissues from animals immunized with Δhfq (a), Wt strain (b), or PBS (c). Scale bars, 100 μm. (TIFF) [file pntd.0005728.s006.tiff]
