# Supplementary material for: An attenuated Shigella mutant lacking the RNA-binding protein Hfq provides cross-protection against Shigella strains of broad serotype
Source: PLoS Negl Trop Dis. 2017 Jul 20;11(7):e0005728. doi: 10.1371/journal.pntd.0005728 (PMC5544247; doi:10.1371/journal.pntd.0005728)
Supplement: S1 Table — (DOCX) [file pntd.0005728.s007.docx]

**S1 Table**. **Bacterial strains used in the study.**

| Bacterial strain | Genotype | Source (References) |
| --- | --- | --- |
| *E. coli* | | |
| BL21 | *E.coli* B strain | New England Biolabs |
| NIID1 | Enteroinvasive *E.coli* clinical isolate | This study |
| *S. sonnei* | | |
| HW383 | *S. sonnei* wild-type strain (Tc^r^) | [26] |
| IDH00968 | *S. sonnei* clinical isolate | This study (NICED) |
| MS390 | HW383 (Tc^s^) | [27] |
| MS1632 | MS390 *ΔinvE* | [27] |
| MS2834 | MS390 *ΔipaA*~*spa40*(*Δ*T3SS) | This study |
| *S. flexneri* |  |  |
| 9268N | *S. flexneri* 1b wild-type strain | [S1 Ref. 1] |
| 9268N17-1 | 9268N lacking the virulence plasmid | [S1 Ref. 1] |
| 2457T | *S. flexneri* 2a wild-type strain | [S1 Ref. 2] |
| MF4835 | 2457T *Δhfq::aphA* | [27] |
| MF4837 | MF4835 carrying pACYC*-ipaBCDA* | This study |
| MF1632 | 2457T *ΔinvE* | This study |
| GTC-01924 | *S. flexneri* 3a wild-type strain | This study (Gifu university) |
| GTC-01927 | *S. flexneri* 6 wild-type strain | This study (Gifu university) |
| *S. dysenteriae* type 1 (*Sd1*) | | |
| NT4907 | *Sd1* wild-type strain | This study (NICED) |
| TSH-1669  MD506 | *Sd1* wild-type strain  *Sd1* cured of virulence plasmid | This study  This study |
|  |  |  |

**S1 Reference**

1. Shaikh N, Terajima J, Watanabe H. IpaC of *Shigella* binds to the C-terminal domain of beta-catenin. Microb Pathog. 2003;35(3):107-17. Epub 2003/08/21. doi: S0882401003000937 [pii]. PubMed PMID: 12927518.

2. Wei J, Goldberg MB, Burland V, Venkatesan MM, Deng W, Fournier G, et al. Complete genome sequence and comparative genomics of *Shigella flexneri* serotype 2a strain 2457T. Infect Immun. 2003;71(5):2775-86. PubMed PMID: 12704152.
