# Supplementary material for: An attenuated Shigella mutant lacking the RNA-binding protein Hfq provides cross-protection against Shigella strains of broad serotype
Source: PLoS Negl Trop Dis. 2017 Jul 20;11(7):e0005728. doi: 10.1371/journal.pntd.0005728 (PMC5544247; doi:10.1371/journal.pntd.0005728)
Supplement: S2 Table — (DOCX) [file pntd.0005728.s008.docx]

**S2 Table.** **Primers used in the study.**

| invE-*Δ*1 | 5'-GCATTTTTTCATCTATGGAGCTCTCACATCAGAGCTCCACAAGAATATTATTCCGGGGATCCGTCGAC-3' |
| --- | --- |
| invE-*Δ*2 | 5'-CGAAGATTTTTATTATCTGAATTGGGCAGTTTACATCAGTGTTCGATGTTTATGTAGGCTGGAGCTGCTTCG-3' |
| invE-verif1 | 5'-AGCTCCACAAGAATATTATTCTTTTATCC-3' |
| invE-verif2 | 5'-CACCAGATAATGTTCTCGTGGTCAGC-3' |
| ipaA/spa40-*Δ*1 | 5'-AATCCTTATTGATATTCTTTAATACTTTTGATAGGGAAGTGGTTACATCTGTAGGCTGGAGCTGCTTCG-3' |
| ipaA/spa40-*Δ*2 | 5'-AATCCTTATTGATATTCTTTAATACTTTTGATAGGGAAGTGGTTACATCTGTAGGCTGGAGCTGCTTCG-3' |
| ipaA/spa40-verif1 | 5'-GTTTGCTGTACGCTATACCATAGC-3' |
| ipaA/spa40-verif2 | 5'-ATTATGGTCAGTCGTTTTTGCTGTC-3' |
